# Borderline personality disorder features and their relationship with trauma and dissociation in a sample of community health service users

**DOI:** 10.1186/s40479-023-00228-x

**Published:** 2023-07-03

**Authors:** Hong Wang Fung, Ming Yu Claudia Wong, Stanley Kam Ki Lam, Emily Nga Man Wong, Wai Tong Chien, Suet Lin Hung, Kun-Hua Lee, Jialiang Cui, Colin A. Ross

**Affiliations:** 1 Department of Social Work, Hong Kong Baptist University, Kowloon, Hong Kong; 2 Department of Health and Physical Education, The Education University of Hong Kong, Ting Kok, Hong Kong; 3 Nethersole School of Nursing, Faculty of Medicine, The Chinese University of Hong Kong, Shatin, Hong Kong; 4 Department of Counselling and Psychology, Hong Kong Shue Yan University, North Point, Hong Kong; 5 Department of Educational Psychology and Counseling, National Tsing Hua University, 521 Nan-Da Road, Hsinchu City, 30014 Taiwan; 6 Department of Social Work, The Chinese University of Hong Kong, Shatin, Hong Kong; 7 The Colin A. Ross Institute for Psychological Trauma, Richardson, TX USA

**Keywords:** Borderline personality disorder (BPD), Dissociation, Trauma, Comorbidity, Trauma-informed care

## Abstract

**Background:**

Previous studies have indicated that borderline personality disorder (BPD) is closely associated with trauma and dissociation. Nevertheless, BPD is a heterogeneous condition, and not all people with BPD have severe dissociation. This study examined whether the relationship of BPD features with trauma and dissociation would remain significant after controlling for some general non-specific mental health distress. We also made the first attempt to explore which specific BPD features would be particularly associated with dissociation.

**Methods:**

We analyzed survey data from a sample of community health service users in Hong Kong (*N* = 376). Hierarchical multiple regression and data-driven network analysis were used.

**Results:**

The lifetime prevalence of DSM-5 BPD was 16.0% in our sample. Of participants who met criteria for BPD, 43.3% scored above cutoff on the dissociation measures, thus possibly having clinically significant dissociative symptoms. BPD features were associated with adulthood trauma and psychoform dissociation even after controlling for age, depression and self-esteem. Network analysis showed that some BPD features – including impulsivity, identity disturbance and suicidal/self-mutilation behaviors – were particularly associated with dissociation; other BPD features such as interpersonal-related problems had relatively weak to no connection with dissociation.

**Conclusions:**

Our results suggested that some particular BPD features might be dissociative in nature, although further longitudinal research is required. We argue that a trauma-informed perspective should be employed when working with clients presenting with BPD features despite these features being commonly stigmatized. Further research on the intervention needs of the people with BPD who suffer from high levels of dissociation is required.

**Supplementary Information:**

The online version contains supplementary material available at 10.1186/s40479-023-00228-x.

## Introduction

Borderline personality disorder (BPD) has been recognized as an official mental disorder since 1980 in the DSM-III [1]. In the DSM-5, BPD is classified as a personality disorder which is characterized by “a pervasive pattern of instability of interpersonal relationships, self-image, and affects, and marked impulsivity” [2]. Although an alternative model has been proposed to diagnose BPD, the criteria-based diagnostic model is still most commonly used [3]. BPD is of public health and clinical significance because it has a lifetime prevalence of about 5.9% in the general population while up to 20% of outpatients and 50% of inpatients meet the criteria for BPD [4, 5]. Patients with BPD typically have long-term psychosocial and occupational impairments and high demands for health and social care resources [6, 8], therefore, BPD is often considered to be a severe mental health condition. BPD is probably one of the most stigmatized mental disorders because people with BPD are often labeled as being manipulative, problematic, attention-seeking and non-compliant [9, 10]. Even mental health professionals sometimes have negative attitudes towards this group of service users [11]. We believe that one important reason for the stigma against people with BPD features is that these features and their etiological factors and the biopsychosocial mechanisms behind the disorder are not well understood.

Many studies have been done to explore the causes of BPD features. As revealed in a systematic review [5], current longitudinal studies have identified a number of psychosocial risk factors for BPD features, such as low socioeconomic status, family and school stressors, childhood adversities, parent/family psychopathology, maltreatment and other traumatic events, insecure attachment, and low IQ during childhood. Many of these risk factors are not BPD-specific but are common risk factors for other mental disorders, especially trauma-related disorders [12]. Given the close relationship between childhood trauma and BPD, it has long been argued that BPD may be better conceptualized as a trauma disorder [13]. Moreover, although BPD and complex post-traumatic stress disorder (CPTSD) are considered to be distinct disorders, the symptoms of these two disorders greatly overlap [14], with dissociation being an important factor in both disorders [15]. As noted, there are many psychosocial risk factors for BPD. Trauma and dissociation are not the only factors. However, from our perspective, trauma and dissociation may help explain some of the BPD features in some cases, as will be further discussed.

Dissociation is generally defined as a disruption or discontinuity in the normal integration of certain parts of the personality, such as emotions, memories, motor controls, and identities [2, 16]. Dissociation is a transdiagnostic phenomenon that can be found in people with different mental disorders [17], including BPD [18]. In the DSM-5, the presence of transient, stress-related dissociative symptoms is one of the diagnostic criteria for BPD. Empirical studies have indicated that people with BPD have higher levels of dissociation than those with other mental disorders, except for PTSD and dissociative disorders (DDs) [19]. DDs also commonly co-occur with BPD, with 37% to 72.5% of patients with BPD meeting criteria for DDs [20, 22] and with 63.7% to 84.3% of patients with dissociative identity disorder (DID) meeting criteria for BPD [22, 23]. In line with this empirical literature, some scholars have proposed that BPD may lie in the middle of the spectrum of trauma-related dissociation, in which simple PTSD involves less severe dissociation, and DID involves the most severe form of dissociation [24, 25]. It should be noted that, like other mental health conditions (e.g., depression and psychosis), BPD is a complex and heterogeneous condition. There may be a subgroup of people with BPD suffering from trauma-related dissociation. In some – although not all –cases, some BPD features may be partly or fully explained by dissociative processes. For example, as observed in some patients with severe DDs, impulsivity or affective instability may sometimes be partly explained by intrusions from or conflicts among dissociated self-states (e.g., the intrusions of an emotionally unstable part makes the host personality state have mood swings or unwanted impulses) [26], although there is a lack of data in this regard. For example, Fisher said that impulses in the context of chronically traumatized patients should be understood as “communications from the trauma-related parts” [27] (p. 13) – in other words, impulses in some cases might be better explained by dissociation of the personality. However, as emphasized again, not only may dissociation be a heterogeneous phenomenon among people with BPD [19], but BPD itself is also a highly heterogeneous condition [28]. According to the DSM-5 classification system, in which five out of nine criteria are required to make a BPD diagnosis, there are 256 possible combinations of BPD criteria. Two people with BPD may share only one out of nine criteria for the disorder. Moreover, as discussed above, many but not all people with BPD suffer from dissociative pathology, suggesting that not all BPD symptoms are equally connected to dissociation. Therefore, the relationship of BPD features with trauma and dissociation requires further investigation in order to better understand which specific BPD features would be particularly related to dissociation. Such knowledge is important to make sense of the BPD features observed in clinical settings and so as to destigmatize people with BPD features. Such knowledge would also be important to reveal whether it would be preferable to encourage a trauma-informed perspective to facilitate recovery when working with service users suffering from BPD features.

This study aimed to contribute to the literature on the relationship between trauma/dissociation and BPD by answering the following research questions. First, we wanted to examine whether the relationship of BPD features with trauma and dissociation would remain significant after controlling for some more general, non-trauma-specific mental health distress, which was operationalized as depression and self-esteem scores in this study. Depressive symptoms are the most common mental health problems in the community, and self-esteem is closely related to mental well-being [29], and both are not specific trauma-related problems. It should be noted that depressive symptoms and self-esteem are two closely related but not the same construct [30]. Moreover, although depressive symptoms are the most common mental health problems, the limitation of not controlling for other less common non-trauma-specific mental health distress (e.g., substance abuse, eating disorders) in the present study should be acknowledged. Second, we wanted to explore which specific BPD features would be particularly associated with dissociative symptoms by using data-driven network analysis. In addition to these two primary research questions, this study also provided updated data regarding the prevalence of BPD in Hong Kong, which is a city with a mix of both Chinese and Western cultures. In particular, the lifetime prevalence of DSM-5 BPD in a sample of community health service users in Hong Kong would be reported because no updated data regarding the prevalence of BPD in Hong Kong is available and because we are not aware of any study investigating BPD in primary care or community health service settings in the Chinese context.

## Methods

### Participants

This study analyzed data from a project that investigated mental health problems among people receiving services from Registered Chinese Medicine Practitioners in the community of Hong Kong. Part of the data (e.g., frequency and sociocultural correlates of post-traumatic symptoms in this sample) not directly relevant to the focus of the present study (i.e., the symptom-level relationship between BPD features and dissociative symptoms) have been reported elsewhere [31]. Registered Chinese Medicine Practitioners are officially recognized service providers who have been specifically trained in traditional Chinese medicine (TCM). They can be regarded as alternative medicine service providers, but TCM services are commonly used by people in Chinese cultures to promote well-being and manage a variety of health problems. TCM service is also regarded as part of primary care services in many Chinese communities; the utilization rate in Hong Kong was reported to be 45.2% [32]. During March to June 2022, we recruited potential participants in local traditional Chinese medicine clinics and through social networking sites using online advertising. The recruitment poster emphasized that this was a health survey study in order to limit potential self-selection bias. Participants had to be aged between 18 to 64, agree to give written informed consent and participate, have received services from any Registered Chinese Medicine Practitioner in the past 3 months, and be able to access and complete the online survey. At the beginning of the online survey, if participants reported that they had been diagnosed with a learning or reading disorder, dementia, and/or cognitive impairments, they were excluded. This study obtained ethics approval at the Chinese University of Hong Kong. This research was conducted in accordance with the Helsinki Declaration as revised 1989.

### Measures

In the online survey, participants were invited to answer some questions on their demographic and health backgrounds (e.g., age, gender, use of mental health services, clinical diagnosis [if any]). The surveys also included well-validated measures of childhood and adulthood trauma, BPD symptoms, dissociative symptoms, depression, and self-esteem.

*Childhood and adulthood trauma* were assessed using the Brief Betrayal Trauma Survey (BBTS), which is a 24-item self-report measure with good test–retest reliability over three years [33]. An example item is “You were deliberately attacked that severely by someone with whom you were very close.” Participants could select “Never”, “1 to 2 times” or “more than that” for each type of experience. They were regarded as having experienced certain specific traumatic events if they endorsed “1 to 2 times” or “more than that” for that item. The Chinese version of the BBTS also had acceptable test–retest reliability with an average agreement of 90.7% between two tests over one week [34]. In this study, we focused on the number of childhood and adulthood traumatic events (possible range: 0 to 12).

*Borderline personality disorder (BPD) features* were assessed using the BPD Section of the Self-Report Dissociative Disorders Interview Schedule (SR-DDIS-BPD). The DDIS is a well-validated structured interview for dissociative disorders, and it also includes sections that assess clinically relevant disorders and symptoms, including BPD [35]. This section includes nine items taken verbatim from DSM-5 that assesses the nine diagnostic criteria for BPD in DSM-5. The response options include “yes”, “no” and “unsure”, with “yes” indicating a positive response for each specific BPD feature. According to the DSM-5 classification system, one must endorse at least five items to meet the DSM-5 BPD criteria. The Chinese version of the SR-DDIS-BPD was found to have excellent convergent validity (*r* = 0.792 with another BPD measure) and satisfactory diagnostic validity (sensitivity = 95.2%, specificity = 64.9%) in a psychiatric sample [36]. Moreover, the Chinese version of the SR-DDIS-BPD had good test–retest reliability over one week in a sample of Chinese young adults (*N* = 116) (total score ICC = 0.842, *p* < 0.001; Cohen’s kappa for each item ranged from 0.44 to 0.79, *p* < 0.001, with a mean Cohen’s kappa of 0.58 [SD = 0.098]) (unpublished data of [34]).

*Psychoform dissociation* was assessed using the Dissociative Identity Disorder (DID) Features of the SR-DDIS (SR-DDIS-DF) [35, 37]. This section includes 16 items that assess features associated with severe DDs (e.g., awareness of another person existing inside, different handwriting styles, blank spells, voices coming from inside). An example item is “Do you ever have blank spells or periods of missing time that you can’t remember, not counting times you have been using drugs or alcohol?” On each item, participants could select “yes”, “no’ or “unsure”, while some items had alternative response options, including “never”, “occasionally”, “fairly often”, and “frequently”. Participants were regarded as having the specific dissociative experience if they selected “yes” or “fairly often”/ “frequently” [35]. This section performs very well in differentiating patients with severe DDs from other psychiatric groups [38]. In the Chinese context, the SR-DDIS-DF was highly correlated with other dissociation measures (*r* = 0.613 to 0.626), and it performed even better than other dissociation screening tools in detecting DDs [39].

*Somatoform dissociation* was assessed with the 5-item Somatoform Dissociation Questionnaire (SDQ-5), which is a reliable and valid shortened version of the original 20-item SDQ [40, 41]. The SDQ assesses how often the participant had a given somatoform experience in the past year (1 = this applies to me NOT AT ALL, 5 = this applies to me EXTREMELY). The item score was maximized to one point if a physical cause of a given experience/symptom was reported [42]. The Chinese version of the SDQ-5 also has good reliability and satisfactory validity [39].

*Depressive symptoms* were assessed using the Patient Health Questionnaire-9 (PHQ-9), which is a 9-item commonly-used measure of depression [43, 44]. It assesses depressive symptoms in the past two weeks (0 = not at all, 3 = nearly every day). The Chinese version of the PHQ has also been validated [45].

*Self-esteem* was assessed using the single-item measure of self-esteem (SISE), which asks, “how satisfied are you with yourself?” (1 = very dissatisfied, 9 – very satisfied) [46]. Robins, Hendin [47] indicated that single-item measures of self-esteem could measure self-esteem very well. The Chinese version of the SISE has moderate test–retest reliability (ICC = 0.815, *p* < 0.001) and construct validity with depression (*r* = -0.571, *p* < 0.001) and fear of negative emotions (*r* = -0.324, *p* < 0.001) (unpublished data of [34]).

### Data analysis

SPSS 22.0 was used to conduct the descriptive and correlation analyses regarding the prevalence of DSM-5 BPD (i.e., number of SR-DDIS-BPD ≥ 5) and the Pearson correlation of BPD features with trauma and dissociation. We then conducted a hierarchical multiple regression analysis to examine whether BPD features would be associated with trauma and dissociation after controlling for other more general non-trauma-specific mental health distress (i.e., depression and self-esteem). After that, the R Version 4.0 software was used to conduct a network analysis of the BPD items with psychoform and somatoform dissociative symptoms. The pairwise Mixed Graphical Model (MGM), which involves both binary and continuous data, was performed in the network analysis. The hyper-parameter γ was set at 0.25 as defaulted by Extended Bayesian Information Criterion (EBIC) "EBIC". Exploratory network analysis was used to reveal the underlying structure of the data on a data-driven basis without assuming prior relationships between variables. Statistical network analysis is a statistical method that examines a network theory that tries to conceptualize and describe the relationships between outcomes and factors of outcomes; psychological symptoms are commonly examined using network analysis [48]. Edges and nodes form a standard cross-sectional network analysis. Nodes refer to the variables being connected in the network, in which the relationships between nodes are examined using partial correlation by controlling for all other variables (nodes) in the network. The partial correlation coefficients are revealed in the lines of the network which are called edges [49]. The case-dropping Bootstrapping (1000 bootstraps) was used to assess the network's accuracy and robustness (stability); while the non-parametric bootstrapping (1000 bootstraps) was used to run difference tests on edge weights. The qgraph program was used to visualize the MGM networks; the quality of the MGM network was examined based on the correlation stability coefficient (CS), predictability (R^2^) and the nodewise error of the nodes [50]. The default correlation cutoff of 0.7 was used as the CS-coefficient criteria in the current network analysis, in order to identify the maximum proportion of cases that could be dropped without compromising the accuracy and validity of the results. It is worth noting that the reflection of level could be adjusted by the researcher yet CS—coefficient should not be below 0.25 [49]. The level of interaction between the variables would also be shown by the weight of the edges between the parameters.

## Results

### Sample characteristics and prevalence of BPD

During March to June, 2022, we received 381 responses to the online survey. Five responses were removed due to duplication or an invalid response to the validity check item (4 + 3 = ?). A total of 376 participants who met all inclusion criteria were included for analysis. Their ages ranged from 18 to 64 (M = 40.48; SD = 12.59). Participants were from all 18 districts of Hong Kong (1.1% to 12% from each district). Most of them were female (80.9%), full-time employed (63.6%), and had a bachelor’s degree (52.9%). Only 15.4% of them were currently seeking professional services for psychological issues. One participant reported a clinical diagnosis of BPD, and none reported a DD diagnosis. The sample characteristics are reported in Table 1 and the prevalence of dissociative symptoms has been further reported elsewhere [51].

**Table 1:** Hierarchical multiple regression predicting borderline personality disorder (BPD) features (*N* = 376)

	Model 1		Model 2		Model 3		Model 4	
Variables	β	p	β	p	β	p	β	p
Constant		.000		.496		.379		.486
Age	-.192	.000	.030	.454	.010	.794	.011	.774
Self-esteem			-.136	.006	-.140	.004	-.136	.004
Depression			.599	.000	.524	.000	.453	.000
Childhood trauma					.031	.516	-.006	.902
Adulthood trauma					.137	.007	.100	.041
Psychoform dissociation							.227	.000
Somatoform dissociation							.021	.614
R^2^	0.037		0.47		0.49		0.533	
Adjusted R^2^	0.034		0.466		0.483		0.524	
F	14.319***		110.012***		71.146***		60.018***	
ΔR^2^	.037		.433		.020		.043	
ΔF	14.319***		152.075***		7.278**		16.905***	

^**^ p = .001 *** p < *.001*

In this sample, participants reported an average of 1.85 (SD = 2.30) (range = 0 to 9) BPD features on the SR-DDIS-BPD, and 16.0% met the DSM-5 criteria for BPD (i.e., SR-DDIS-BPD ≥ 5). The frequency of each BPD feature is reported in Table 2. In addition, among participants who met the DSM-5 criteria for BPD (*n* = 60), 13.3% had clinically significant psychoform dissociation (SR-DDIS-DF ≥ 5) while 41.7% had clinically significant somatoform dissociation (SDQ ≥ 9); 43.3% had either or both psychoform and somatoform dissociation. In this subsample (*n* = 60), 76.6% and 93.3% reported at least one type of childhood and adulthood trauma, respectively.

**Table 2:** Frequency of BPD features and the summary of predictability of nodes

Items	Frequency	Percentage of explained variance(R^2^)	Character Vector nodewise Accuracy	Accuracy of the intercept/marginal model
BPD1 Impulsivity	23.4%	/	0.85	0.76
BPD2 Unstable/intense interpersonal relationships	22.1%	/	0.85	0.78
BPD3 Intense/uncontrollable anger	20.2%	/	0.84	0.79
BPD4 Identity disturbance	15.4%	**/**	0.89	0.84
BPD5 Affective instability	37.0%	/	0.81	0.62
BPD6 Frantic efforts to avoid abandonment	19.1%	/	0.86	0.80
BPD7 Suicidal/self-mutilation behaviors	3.5%	/	0.97	0.96
BPD8 Chronic emptiness	31.4%	/	0.75	0.68
BPD9 Stress-related paranoia or dissociation	12.8%	/	0.88	0.86
Somatoform dissociation		0.24	/	/
Psychoform dissociation		0.39	/	/

### The association of BPD features with trauma and dissociation

We first report the correlations of BPD features with other major variables: The number of BPD features (i.e., the severity of the overall BPD presentation) was correlated with age (r = -.192, *p* < .001), childhood trauma (r = .311, *p* < .001), adulthood trauma (r = .410, *p* < .001), depression (r = .677, *p* < .001), self-esteem (r = -.513, *p* < .001), psychoform dissociation (r = .490, *p* < .001) and somatoform dissociation (r = .354, *p* < .001). Independent sample t tests revealed that the number of BPD features was not associated with gender (*p* = .934), a bachelor’s degree (*p* = .692) or full-time employment (*p* = .934). In addition, psychoform dissociation was positively correlated with childhood trauma (r = .343, *p* < .001), and adulthood trauma (r = .361, *p* < .001); somatoform dissociation was also positively correlated with childhood trauma (r = .225, *p* < .001), and adulthood trauma (r = .344, *p* < .001).

Further analysis using hierarchical multiple regression indicated that, after controlling for age, self-esteem and depression, the relationship between adulthood trauma and BPD features remained significant (β = 0.137, *p* = 0.007) (Model 3). The addition of dissociative symptoms to the prediction of BPD features (Model 4) led to a statistically significant increase in R2 of 0.043, F = 60.018, *p* < 0.001. In this model, psychoform dissociation was the second strongest predictor of BPD features (β = 0.227, *p* < 0.001), even after taking the effects of age, self-esteem, depression and trauma into account. The findings are summarized in Table 1.

### The estimated network

**Fig. 1:**
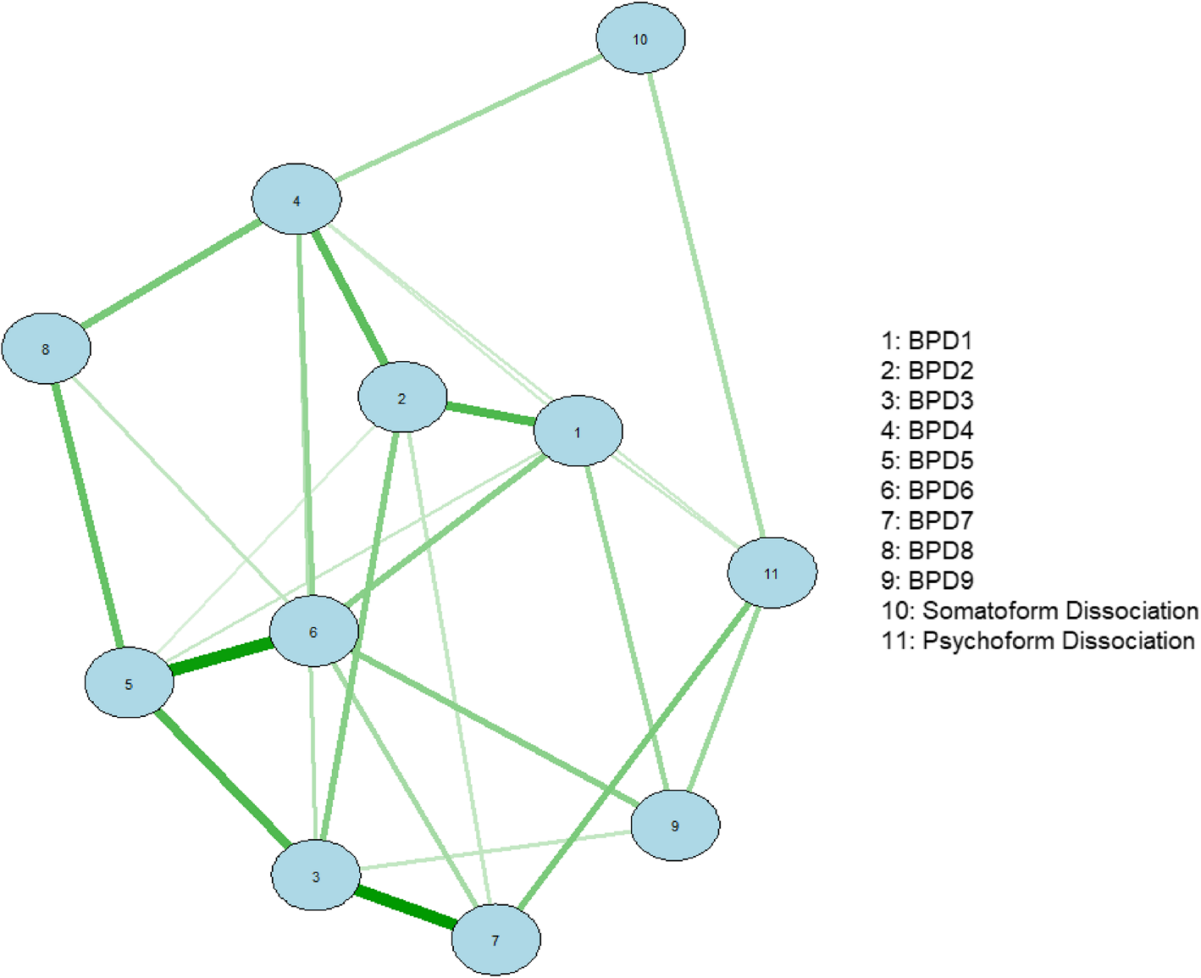
The Estimated Network

**Fig. 2:**
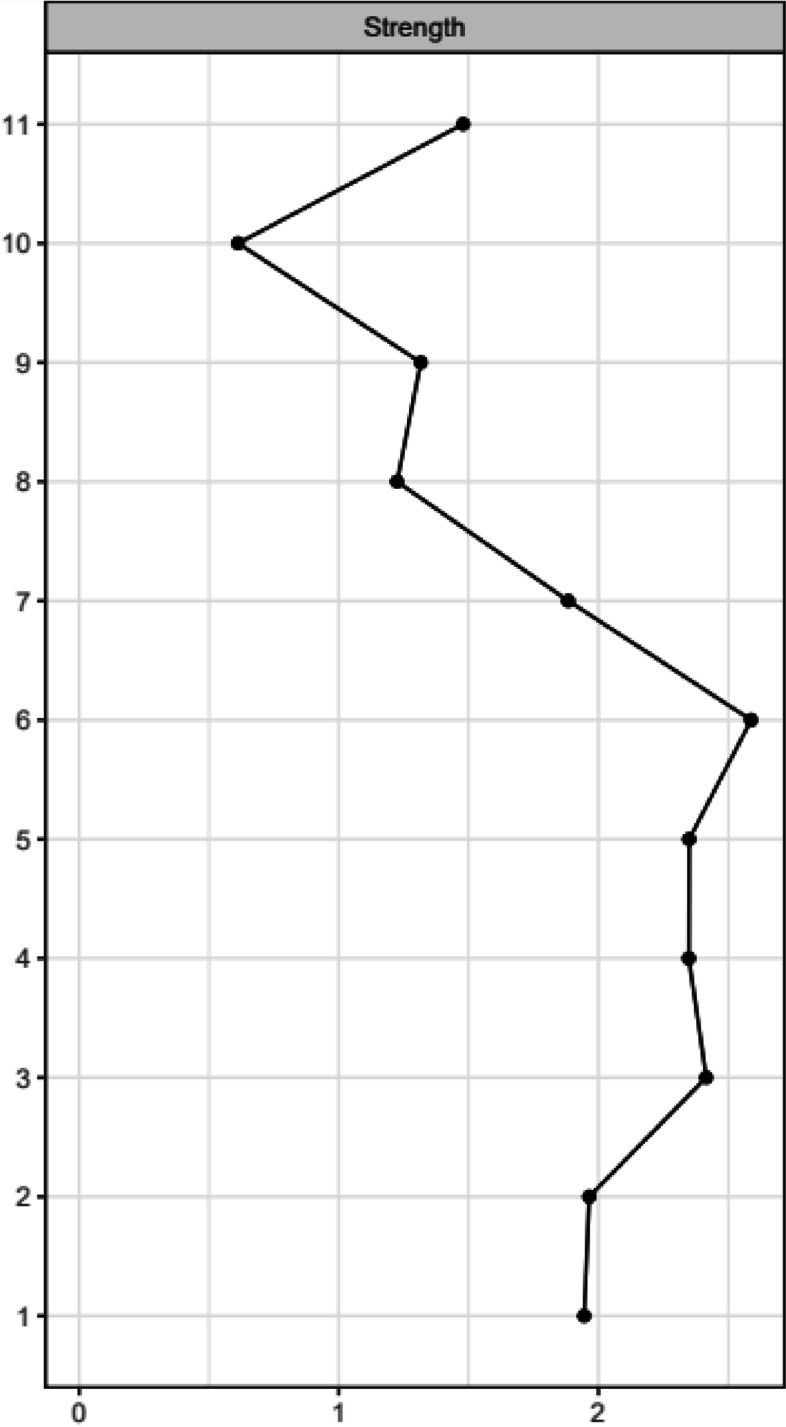
Centrality Plot

**Fig. 3:**
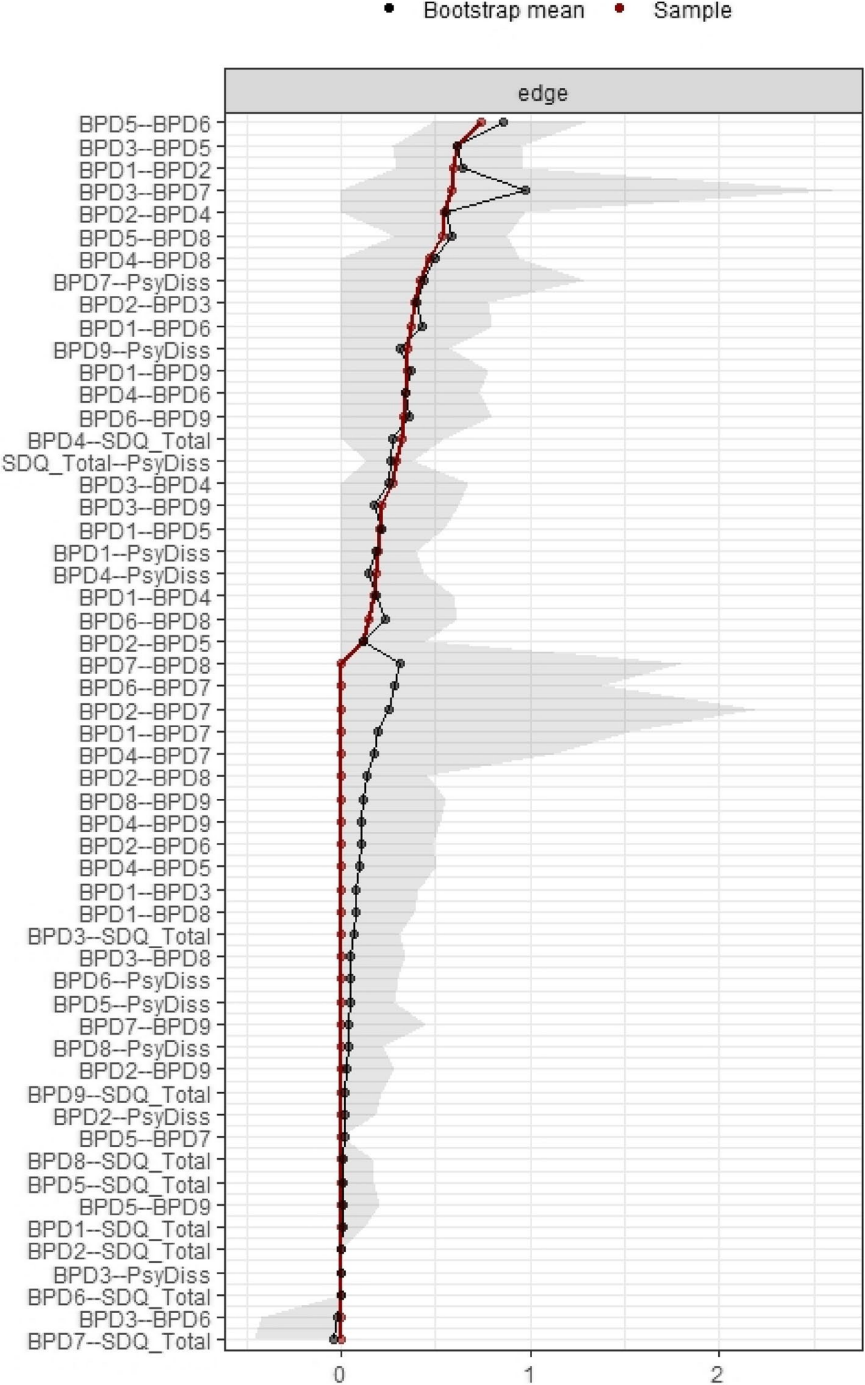
The Edge Weight Stability Plot

Network analysis was conducted to explore the viable connections among the BPD items, psychoform and somatoform dissociative symptoms. In order to quantify the quality of the network model, the predictability, stability and accuracy of nodes are performed. There were different types of node predictability measures, especially when dealing either with continuous variables or categorical variables. The prediction errors function from the mgm model were used to generate the nodewise accuracy and intercept model accuracy, and to interpret the nodes predictability of categorical (non-parametric) variables. Based on the network predictability summary (Table 2), the R^2^ of the somatoform and psychoform dissociation were indicated as parametric outcome, with 0 indicating completely unable to forecast by other nodes and 1 suggesting can be accurately predicted by the other nodes [52]. The score of somatoform and psychoform dissociation (the node) was able to predict the nearby nodes in the network to a considerable extent, as indicated by R^2^ of the somatoform and psychoform dissociation ranging at 0.2 to 0.39. The nodewise accuracy and the intercept/marginal model accuracy were shown to reflect, respectively, the proportion of proper classification (accuracy) of the nodes and the intercept model's overall accuracy. According to Table 2, every node indicated a satisfactory accuracy of 0.70 or higher, and the intercept models demonstrated a respectable degree of accuracy of 0.6 or above, both of which confirmed the accuracy of the entire model. Figures 1 and 2 demonstrates the centrality plot of the network. Based on the correlation stability coefficient for node strength (CS) of 0.36 and edge correlation stability coefficient of 0.13, it indicated that only 36% of the dataset could be dropped in order to maintain the same network structure; and 13% of chance to regenerate the edges to form different network structure when replicating the network. Among the nine BPD items (see Table 3), the strongest edge was found between BPD item 7 (suicidal/self-mutilation behaviors) and psychoform dissociation (interaction-weight = 0.46). Item 9 (stress-related paranoia or dissociation; interaction-weight = 0.350), item 1 (impulsivity; interaction-weight = 0.19) and item 4 (identity disturbance; interaction weight = 0.18) also demonstrated a low to moderate connection with psychoform dissociation. The non-parametric bootstrapping difference test indicated no significant differences between the edge weight connecting psychoform dissociation to BPD item 1, item 4, item 7 and item 9 (See Supplementary Material Figure 1 and 2). Table 3 shows the interaction weighting between each of the items. By interpreting the regression on the categorical variables, the increasing of “psychoform dissociation” by one unit would increase the probability of getting a “Yes” in BPD item 1, item 4, item 7 and item 9. However, it is being seen that the probability of getting a “Yes” in item 7 and item 9 are higher than that in item 1 and item 4. For somatoform dissociation, the strongest connection was with BPD item 4 (identity disturbance; interaction-weight = 0.32). Similarly, the interpretation of regression on categorical variable –BPD item 4 indicated that a higher level of “somatoform dissociation” increases the probability of having a “Yes” in BPD item 4.The other items did not show significant edges with psychoform dissociation and somatoform dissociation in the Mixed Graphical Network (see Table 3).In order to demonstrate the accuracy of the edge weight in the non-parametric bootstrap test, the edge weight accuracy stability was shown Fig. 3. Figure 3 shows the edge estimate of the current sample, the 95% confidence interval band derived from the bootstrapped edge weights and the mean edge estimate in the bootstrapped data. Visually, some of the sample edge weights are more accurate than others. However, there are some intervals that do not coincide with the bootstrapped mean edge, which suggests a certain amount of variability in the estimation of edge weights if the study is replicated.

**Table 3:** The interaction weighting in the mixed graphical models

	BPD1	BPD2	BPD3	BPD4	BPD5	BPD6	BPD7	BPD8	BPD9	Somatoform dissociation	Psychoform dissociation
BPD1 Impulsivity	1										
BPD2 Unstable/intense interpersonal relationships	0.63	1									
BPD3 Intense/uncontrollable anger	0.00	0.43	1								
BPD4 Identity disturbance	0.17	0.57	0.27	1							
BPD5 Affective instability	0.20	0.14	0.61	0.000	1						
BPD6 Frantic efforts to avoid abandonment	0.41	0.00	0.00	0.37	0.86	1					
BPD7 Suicidal/self-mutilation behaviors	0.00	0.21	0.89	0.000	0.000	0.31	1				
BPD8 Chronic emptiness	0.00	0.00	0.00	0.47	0.54	0.22	0.00	1			
BPD9 Stress-related paranoia or dissociation	0.35	0.00	0.00	0.000	0.000	0.41	0.00	0.00	1		
Somatoform dissociation	0.00	0.00	0.00	0.32	0.000	0.00	0.00	0.00	0.000	1	
Psychoform dissociation	0.19	0.00	0.00	0.18	0.000	0.00	0.46	0.00	0.350	0.29	1

## Discussion

This study contributes to the limited literature on the relationship between BPD features, trauma and dissociation. We have also provided the first data regarding the prevalence of DSM-5 BPD (16.0%) among community health service users in Hong Kong. The major findings include: 1) many but not all participants with BPD scored above the cut-off on the dissociation measures (i.e., indicating clinically significant dissociative symptoms) (43.3%); 2) the severity of the overall BPD presentation (measured as the number of BPD features) was significantly associated with trauma and dissociation after controlling for age and other some common non-trauma-specific mental health distress (including depressive symptoms and self-esteem); and 3) data-driven analysis revealed that some but not all BPD features were associated with dissociative symptoms. These results and their implications require further discussion.

First of all, network analysis suggested that only some BPD features were associated with dissociative symptoms based on the MGM. These included suicidal/self-mutilation behaviors, identity disturbance, impulsivity, and stress-related paranoia or dissociation (see Table 3). The fact that 9^th^ BPD symptom (i.e., stress-related paranoia or dissociation) correlated with dissociation as measured by the SR-DDIS is unremarkable; our focus is on the relationship between dissociation and other BPD criteria. With respect to suicidal/self-mutilation behaviors, our findings are consistent with previous studies which indicated that dissociation is a strong predictor of self-harm and suicidality in clinical settings [53, 54], including in patients with BPD [55]. The complex relationship between dissociation and self-harm and suicidality is not yet well understood, and the possible reasons behind this relationship require further research, but self-harm and suicidality sometimes may be conceptualized as an attempt to respond to or cope with trauma-related dissociative experiences (e.g., depersonalization, intrusive symptoms) [54]. As observed clinically in patients with trauma and dissociation, sometimes self-harm might also result from the punishment inflicted on certain dissociated self-states by other, hostile self-states (e.g., introjects).

In addition, we found that identity disturbance was particularly associated with dissociative symptoms as well. While identity disturbance is a core domain of BPD, it is also one of the core features of people with severe DD [26]. Therefore, it is reasonable that identity disturbance is associated with dissociative symptoms [18]. Whether or not identity disturbance in BPD can be better conceptualized as the result of structural dissociation [25] remains an interesting yet unanswered question, but our finding indicates the need for further research in this area. Moreover, we also found that impulsivity was particularly associated with dissociation. This BPD feature may also be explained by dissociative processes to a certain degree. For example, impulsivity, in many cases, can be understood as resulting from a conflict between different dissociated parts of self, or as the result of switching of executive control between self-states.

In the most extreme form of dissociation, patients with DID have dissociated identities associated with distinct self-concepts, emotions, memories, interests and behaviors, which could result in patterns corresponding to these BPD features, including identity disturbance and impulsivity. In some cases, impulsivity may also be explained by intrusions of other dissociated parts while the host personality is still in executive control (partial dissociation) [56]. Therefore, while not all people with BPD have a diagnosable DD, some specific BPD features such as self-harm, identity disturbance, and impulsivity may particularly indicate that the person may have difficulties in integrating certain parts of his/her self (i.e., dissociation of the personality) [2, 25]. On the other hand, the present study found that other BPD features, such as interpersonal-related BPD features (i.e., unstable/intense interpersonal relationships and frantic efforts to avoid abandonment) and anger, had no statistically significant connection with dissociation according to our MGM results. The reasons behind this finding require further research and discussion in the future,, despite the fact that both dissociation and these BPD features commonly occur in trauma survivors [57]. Although we did not focus on the relationship between trauma and dissociation in this study, both childhood and adulthood trauma were significantly correlated with dissociation in the dataset, which is consistent with the widely reported relationship between trauma and dissociation in the literature. Nevertheless, it is important to acknowledge the fact that the strong relationship between trauma and dissociation does not imply that all dissociation is trauma-related. In addition, although BPD symptoms were only slightly associated with adulthood trauma (β = 0.100) in the regression model, the correlation analyses indicated that BPD symptoms were positively correlated with both childhood trauma (*r* = 0.311), adulthood trauma (*r* = 0.410). The relatively weak association between trauma and BPD symptoms is unexpected. It implies that the possible moderators behind this relationship in the Chinese context require more research in the future.

In line with the above-mentioned data-driven findings, we found that 43.3% of participants with BPD may have clinically significant dissociative symptoms (indicated by SR-DDIS-DF ≥ 5 or SDQ-5 ≥ 9). In addition, although psychoform dissociation was associated with the severity of the overall BPD presentation after controlling for other general non-trauma-specific mental health distress, the relationship was not very strong (β = 0.227). Taken together, our results indicate that many but not all BPD features may be dissociative in nature. There may be a dissociative subtype of BPD [24], but not all people with BPD are highly dissociative. It should be noted that 76.6% and 93.3% of participants with BPD reported at least one type of childhood and adulthood trauma, respectively.

It is important to employ a trauma-informed perspective to understand the “problematic” behaviors of people with BPD. BPD features related to interpersonal difficulties may result from lack of a role model demonstrating how to maintain healthy interpersonal boundaries; BPD features related to affective instability and impulsivity may result from a lack of healthy coping strategies and integrative capability to manage the dissociated/unprocessed parts of self [25, 58, 59, 60]. If BPD features can be made sense of by recognizing the connections between life experiences and current symptoms, instead of the medical model, a trauma-informed approach can be employed, and more accurate, destigmatizing and non-retraumatizing interventions can be offered. In particular, our results indicate that BPD features such as impulsivity, identity disturbance, or suicidal/self-mutilation behaviors may be helpful indicators of more severe dissociative symptoms. When a client presents with such BPD features, a detailed assessment of dissociation should be considered in order to ensure early identification and timely interventions. Failing to recognize dissociation in people with BPD features may lead to less effective interventions [61]. Nevertheless, further research on the clinical features and treatment needs of people with BPD who exhibit high levels of dissociation is required. Additionally, the differences in treatment needs for BPD patients with high and low levels of dissociation require further investigation.

Although this study made the first attempt to use network analysis to examine the relationship between dissociation and different specific BPD features and although the findings may have important implications for the understanding, assessment and treatment of BPD, the study has several limitations. First, we only recruited participants in one city and most participants were female, therefore the results might have limited generalizability. Second, although our measures are well-validated, we relied on self-report data and did not use structured interviews to confirm the diagnostic status of the participants. Third, the cross-sectional nature of this study did not allow causal inferences to be drawn concerning the variables – importantly, although we found that some specific BPD features are more strongly associated with dissociative symptoms, further longitudinal studies are required to examine whether dissociation would play a causal role in these BPD features. Fourth, we only controlled for depressive symptoms and self-esteem, but did not control for anxiety symptoms in this study, although anxiety may also be closely associated with dissociation. Finally, Epskamp, Borsboom [62] have questioned the viability of the edge weight significance difference test despite the fact that it was commonly applied in network research due to its extremely high probability of interacting with both Type 1 and Type 2 error and resulting in a low significance level.

### Concluding remarks

This study provides the first data regarding the prevalence of DSM-5 BPD in a sample of community health service users in Hong Kong. We also made the first attempt to use network analysis to explore the relationship between dissociation and different BPD features. The findings suggest that a considerable subgroup of people with BPD may be suffering from trauma-related dissociation. A trauma-informed perspective should be employed when working with clients presenting with BPD features. Their unstable, rapidly changing “problematic” behaviors can be better understood as responses to trauma and stress, with dissociation being a potentially important underlying issue. This study highlights several BPD features that may be particularly associated with dissociative symptoms, and points to the need for more research on dissociation in people with BPD features.

## Supplementary Information

**Additional file 1:** **Supplementary Figure 1.** Bootstrapping Non-parametric Difference Test. **Supplementary Figure 2.** Bootstrap Non-parametric Difference Test. **Appendix 1.** The R Studio Syntax for the Non-parametric MGM network analysis.

## Data Availability

The data that support the findings of this study are available from the corresponding author on reasonable request.

## References

[1] American Psychiatric Association. Diagnostic and statistical manual of mental disorders (3rd ed). Arlington: Author; 1980.

[2] American Psychiatric Association (2013). Diagnostic and statistical manual of mental disorders.

[3] Vanwoerden S, Stepp SD (2022). The Diagnostic and Statistical Manual of Mental Disorders, alternative model conceptualization of borderline personality disorder: A review of the evidence. Personal Disord Theory Res Treat.

[4] Gunderson JG, Herpertz SC, Skodol AE, Torgersen S, Zanarini MC (2018). Borderline personality disorder. Nat Rev Dis Primers.

[5] Stepp SD, Lazarus SA, Byrd AL (2016). A systematic review of risk factors prospectively associated with borderline personality disorder: Taking stock and moving forward. Personal Disord Theory Res Treat.

[6] Gunderson JG, Stout RL, McGlashan TH, Shea MT, Morey LC, Grilo CM (2011). Ten-year course of borderline personality disorder: psychopathology and function from the Collaborative Longitudinal Personality Disorders study. Arch Gen Psychiatry.

[7] Alvarez-Tomás I, Soler J, Bados A, Martín-Blanco A, Elices M, Carmona C (2017). Long-term course of borderline personality disorder: A prospective 10-year follow-up study. J Pers Disord.

[8] Black DW, Blum N, Pfohl B, Hale N (2004). Suicidal behavior in borderline personality disorder: prevalence, risk factors, prediction, and prevention. J Pers Disord.

[9] Ring D, Lawn S. Stigma perpetuation at the interface of mental health care: a review to compare patient and clinician perspectives of stigma and borderline personality disorder. J Mental Health. 2019:1-21. 10.1080/09638237.2019.1581337. 10.1080/09638237.2019.1581337 30862201

[10] Ahmed S, Newman D, Yalch M (2021). The stigma of borderline personality disorder. Adv Psychol Res.

[11] Sansone RA, Sansone LA (2013). Responses of mental health clinicians to patients with borderline personality disorder. Innov Clin Neurosci.

[12] De Venter M, Demyttenaere K, Bruffaerts R (2013). The relationship between adverse childhood experiences and mental health in adulthood. A systematic literature review. Tijdschr Psychiatr.

[13] MacIntosh HB, Godbout N, Dubash N (2015). Borderline personality disorder: Disorder of trauma or personality, a review of the empirical literature. Can Psychol.

[14] Jowett S, Karatzias T, Shevlin M, Albert I (2020). Differentiating symptom profiles of ICD-11 PTSD, complex PTSD, and borderline personality disorder: A latent class analysis in a multiply traumatized sample. Personal Disord Theory Res Treat.

[15] Ford JD, Courtois CA (2021). Complex PTSD and borderline personality disorder. Borderline Personality Disord Emot Dysregul.

[16] Fung HW, Ross CA, Lam SKK, Hung SL (2022). Recent research on the interventions for people with dissociation. Eur J Trauma Dissociation.

[17] Lyssenko L, Schmahl C, Bockhacker L, Vonderlin R, Bohus M, Kleindienst N (2018). Dissociation in psychiatric disorders: A meta-analysis of studies using the Dissociative Experiences Scale. Am J Psychiatry.

[18] Krause-Utz A (2022). Dissociation, trauma, and borderline personality disorder. Borderline Personal Disord Emot Dysregul.

[19] Scalabrini A, Cavicchioli M, Fossati A, Maffei C (2016). The extent of dissociation in borderline personality disorder: A meta-analytic review. J Trauma Dissociation.

[20] Ross CA (2007). Borderline personality disorder and dissociation. J Trauma Dissociation.

[21] Şar V, Akyuz G, Kugu N, Ozturk E, Ertem-Vehid H (2006). Axis I dissociative disorder comorbidity in borderline personality disorder and reports of childhood trauma. J Clin Psychiatry.

[22] Ross CA, Ferrell L, Schroeder E (2014). Co-occurrence of dissociative identity disorder and borderline personality disorder. J Trauma Dissociation.

[23] Ross CA, Miller SD, Reagor P, Bjornson L, Fraser GA, Anderson G (1990). Structured interview data on 102 cases of multiple personality disorder from four centers. Am J Psychiatry.

[24] Mosquera D, Gonzalez A, Van der Hart O (2011). Borderline personality disorder, childhood trauma and structural dissociation of the personality. Revista Persona.

[25] Van der Hart O, Nijenhuis ER, Steele K (2006). The haunted self: Structural dissociation and the treatment of chronic traumatization.

[26] Steinberg M, Schnall M (2000). The stranger in the mirror: Dissociation—The hidden epidemic.

[27] Fisher J (2014). The treatment of structural dissociation in chronically traumatized patients.

[28] Cavelti M, Lerch S, Ghinea D, Fischer-Waldschmidt G, Resch F, Koenig J (2021). Heterogeneity of borderline personality disorder symptoms in help-seeking adolescents. Borderline Personal Disord Emot Dysregul.

[29] Du H, King RB, Chi P (2017). Self-esteem and subjective well-being revisited: The roles of personal, relational, and collective self-esteem. PLoS ONE.

[30] Sowislo JF, Orth U (2013). Does low self-esteem predict depression and anxiety? A meta-analysis of longitudinal studies. Psychol Bull.

[31] Fung HW, Wong NM, Lam SKK, Chien WT, Hung SL, Ross CA, Cloitre M. Prevalence and sociocultural correlates of post-traumatic stress disorder (PTSD) and complex PTSD among Chinese community health service users in Hong Kong. Int J Soc Psychiatry. 2022. 10.1177/00207640221141018. 10.1177/00207640221141018 36457219

[32] Chiu SWK, Sze T-o (2018). Changes in the utilizationof Chinese medicine in Hong Kong (1993–2015) (in Chinese: 誰在看中醫?香港中醫就診趨勢回顧). Hong Kong J Soc Sci.

[33] Goldberg LR, Freyd JJ (2006). Self-reports of potentially traumatic experiences in an adult community sample: Gender differences and test-retest stabilities of the items in a brief betrayal-trauma survey. J Trauma Dissociation.

[34] Fung HW, Chien WT, Ling HWH, Ross CA, Lam SKK.The mediating role of post-traumatic stress disorder symptoms in the relationship between childhood adversities and depressive symptoms in two samples. Child Abuse Neglect. 2022. 10.1016/j.chiabu.2022.105707. 10.1016/j.chiabu.2022.105707 35714440

[35] Ross CA, Heber S, Norton GR, Anderson D, Anderson G, Barchet P (1989). The Dissociative Disorders Interview Schedule: A structured interview. Dissociation.

[36] Fung HW, Chan C, Lee CY, Yau CKM, Chung HM, Ross CA (2020). Validity of a web-based measure of borderline personality disorder: A preliminary study. J Evid Based Soc Work.

[37] Ross CA, Browning E (2017). The Self-Report Dissociative Disorders Interview Schedule: A preliminary report. J Trauma Dissociation.

[38] Ross CA, Ellason JW (2005). Discriminating among diagnostic categories using the Dissociative Disorders Interview Schedule. Psychol Rep.

[39] Fung HW, Choi TM, Chan C, Ross CA (2018). Psychometric properties of the pathological dissociation measures among Chinese research participants – A study using online methods. J Evid Informed Soc Work.

[40] Nijenhuis ER, Spinhoven P, van Dyck R, Van der Hart O, Vanderlinden J (1996). The development and psychometric characteristics of the Somatoform Dissociation Questionnaire (SDQ-20). J Nerv Ment Dis.

[41] Nijenhuis ER, Spinhoven P, van Dyck R, Van der Hart O, Vanderlinden J (1997). The development of the somatoform dissociation questionnaire (SDQ-5) as a screening instrument for dissociative disorders. Acta Psychiatr Scand.

[42] Nijenhuis ER. The scoring and interpretation of the SDQ-20 and SDQ-5. ANS J Neurocognitive Res. 2010;52(1). 10.1007/BF03379561.

[43] Kung S, Alarcon RD, Williams MD, Poppe KA, Moore MJ, Frye MA (2013). Comparing the Beck Depression Inventory-II (BDI-II) and Patient Health Questionnaire (PHQ-9) depression measures in an integrated mood disorders practice. J Affect Disord.

[44] Kroenke K, Spitzer RL, Williams J (2001). The PHQ-9: Validity of a brief depression severity measure. J Gen Intern Med.

[45] Yeung A, Fung F, Yu S-C, Vorono S, Ly M, Wu S (2008). Validation of the Patient Health Questionnaire-9 for depression screening among Chinese Americans. Compr Psychiatry.

[46] Sawicki–Luiza PAAA, Atroszko SB. Validity and reliability of single-item self-report measure of global self-esteem. London: CER Comparative European Research; 2017.

[47] Robins RW, Hendin HM, Trzesniewski KH (2001). Measuring global self-esteem: Construct validation of a single-item measure and the Rosenberg Self-Esteem Scale. Pers Soc Psychol Bull.

[48] Robinaugh DJ, Hoekstra RHA, Toner ER, Borsboom D (2020). The network approach to psychopathology: A review of the literature 2008–2018 and an agenda for future research. Psychol Med.

[49] Epskamp S, Waldorp LJ, Mõttus R, Borsboom D (2018). The Gaussian Graphical Model in cross-sectional and time-series data. Multivar Behav Res.

[50] Hevey D (2018). Network analysis: a brief overview and tutorial. Health Psychol Behav Med..

[51] Fung HW, Wong ENM, Lam SKK, Chien WT, Hung SL, Ross CA. The prevalence of dissociative symptoms and disorders: Findings from a sample of community health service users in Hong Kong. Asian J Psychiatry. 2022. 10.1016/j.ajp.2022.103351. 10.1016/j.ajp.2022.103351 36495727

[52] Haslbeck JM, Waldorp LJ (2018). How well do network models predict observations? On the importance of predictability in network models. Behav Res Methods.

[53] Foote B, Smolin Y, Neft DI, Lipschitz D (2008). Dissociative disorders and suicidality in psychiatric outpatients. J Nerv Ment Dis.

[54] Calati R, Bensassi I, Courtet P (2017). The link between dissociation and both suicide attempts and non-suicidal self-injury: Meta-analyses. Psychiatry Res.

[55] Wedig MM, Silverman MH, Frankenburg FR, Reich DB, Fitzmaurice G, Zanarini MC (2012). Predictors of suicide attempts in patients with borderline personality disorder over 16 years of prospective follow-up. Psychol Med.

[56] Dell PF, Dell PF, O'Neil JA (2009). The phenomena of pathological dissociation. Dissociation and the dissociative disorders: DSM-V and beyond.

[57] Cloitre M, Courtois CA, Ford JD, Green BL, Alexander P, Briere J, et al. The ISTSS expert consensus treatment guidelines for complex PTSD in adults. 2012. https://istss.org/ISTSS_Main/media/Documents/ComplexPTSD.pdf.

[58] Fisher J. The work of stabilization in trauma treatment. Trauma Center Lecture Series. 1999. http://janinafisher.com/pdfs/stabilize.pdf.

[59] Cloitre M, Cohen LR, Ortigo KM, Jackson C, Koenen KC (2020). Treating survivors of childhood abuse and interpersonal trauma: STAIR Narrative Therapy.

[60] Fung HW, Ross CA. Be a teammate with yourself: Understanding trauma and dissociation. Richardson: Manitou Communications; 2019.

[61] Krause-Utz A, Frost R, Chatzaki E, Winter D, Schmahl C, Elzinga BM (2021). Dissociation in borderline personality disorder: Recent experimental, neurobiological studies, and implications for future research and treatment. Curr Psychiatry Rep.

[62] Epskamp S, Borsboom D, Fried EI (2018). Estimating psychological networks and their accuracy: A tutorial paper. Behav Res Methods.

