# Supplementary material for: Borderline personality disorder features and their relationship with trauma and dissociation in a sample of community health service users
Source: Borderline Personal Disord Emot Dysregul. 2023 Jul 3;10:22. doi: 10.1186/s40479-023-00228-x (PMC10316594; doi:10.1186/s40479-023-00228-x)
Supplement: Supplementary file 1 — Additional file 1: Supplementary Figure 1. Bootstrapping Non-parametric Difference Test. Supplementary Figure 2. Bootstrap Non-parametric Difference Test. Appendix 1. The R Studio Syntax for the Non-parametric MGM network analysis. [file 40479_2023_228_MOESM1_ESM.docx]

Supplementary Figure 1. Bootstrapping Non-parametric Difference Test (Edge)


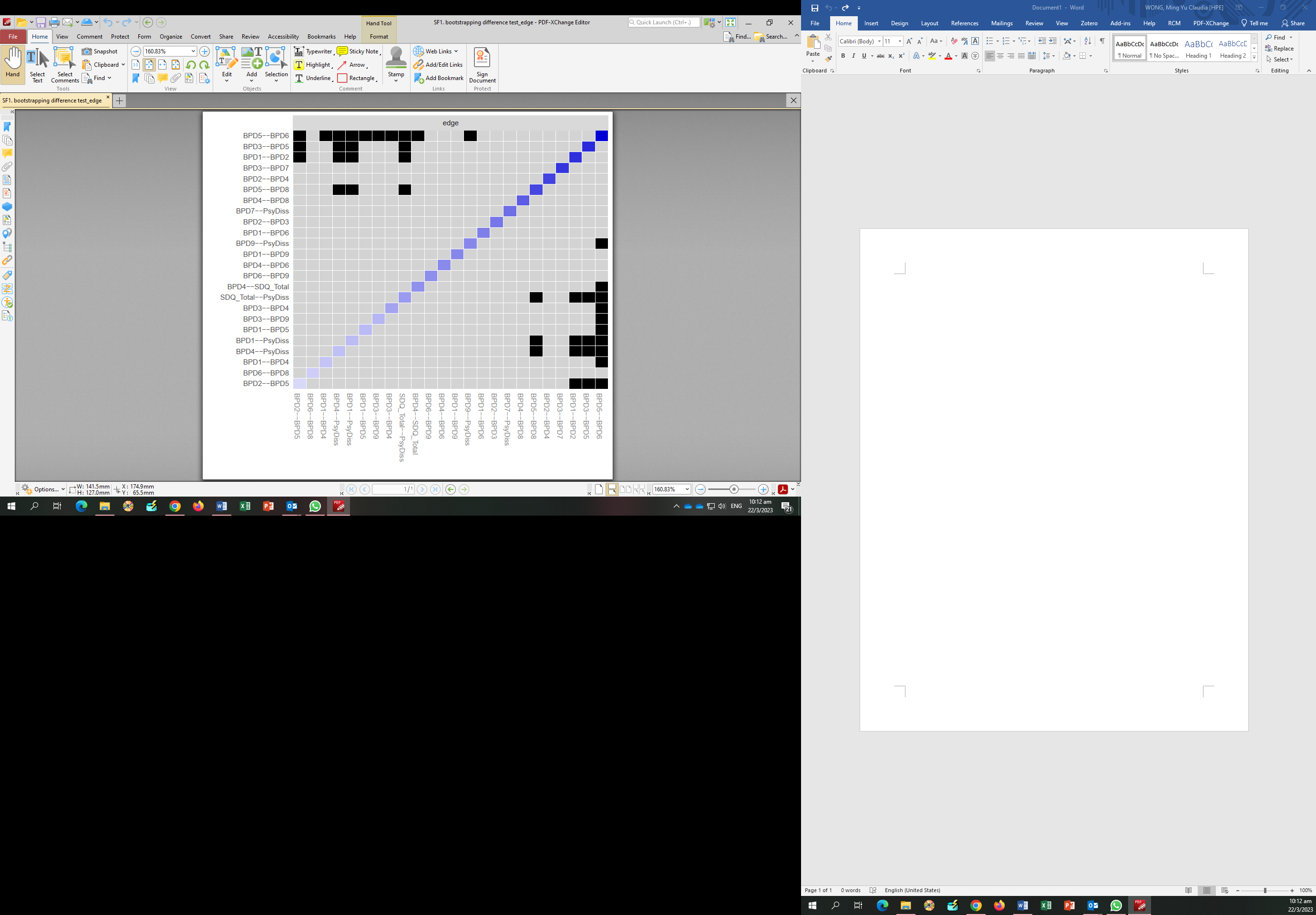


Supplementary Figure 2. Bootstrap Non-parametric Difference Test (Strength)


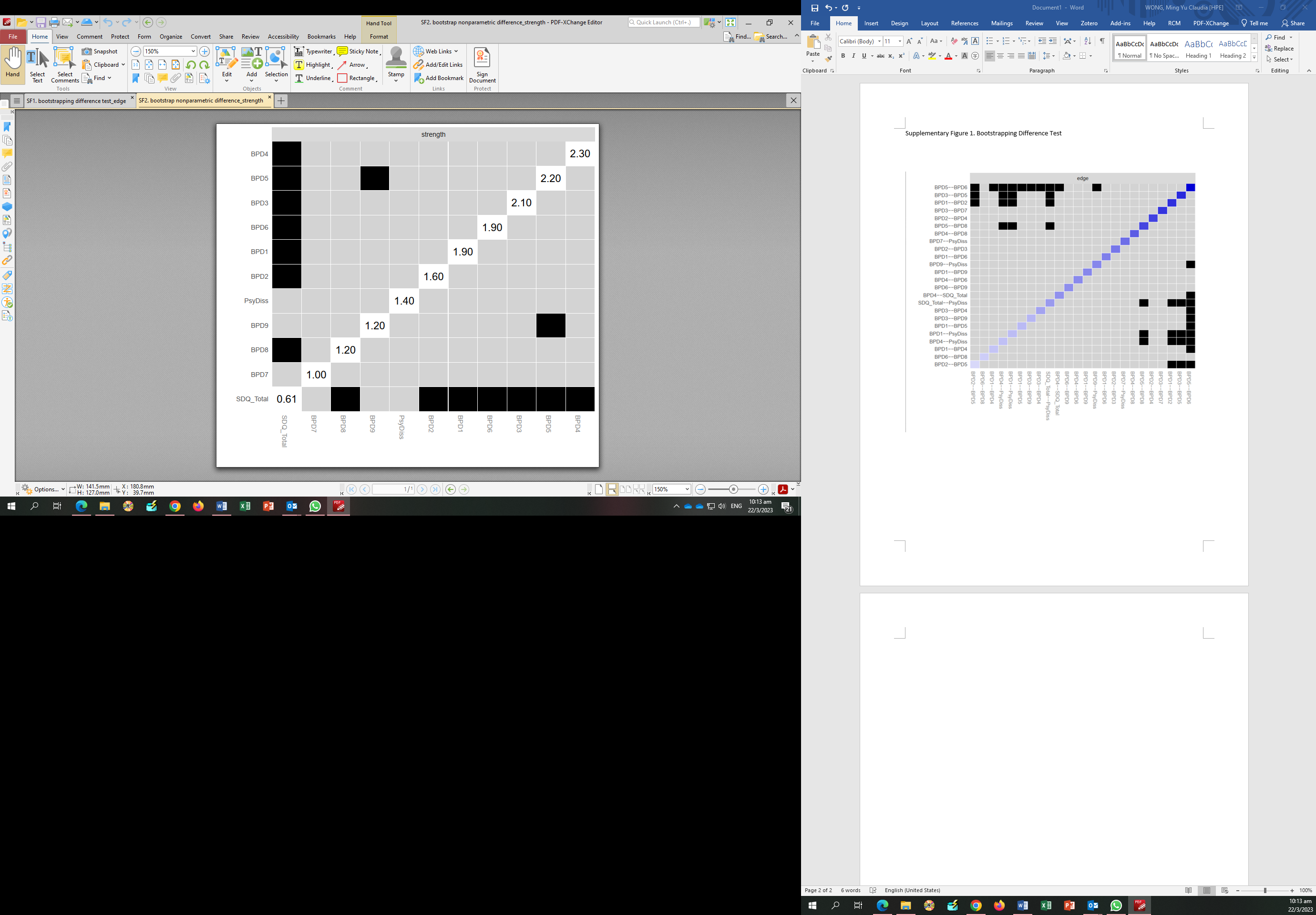


Appendix 1. The R Studio Syntax for the Non-parametric MGM network analysis

**Step 1: Fitting the MGM to the data**

mynetwork <- mgm(data= as.matrix(BPD_and_dissociation), type = “gaussian” ,level =”interval” ,lambdaSel = "EBIC",lambdaGam = 0.25, k = 2, pbar = FALSE, scale = TRUE, ruleReg = "AND", saveData = TRUE, saveModels = TRUE)

**Step 2: Visualizing the Network**

myplot<-qgraph(mynetwork$pairwise$wadj, vsize=6, layout="spring", color="lightblue", edge.colour= mynetwork$pairwise$edgecolor, border.width=1.5,border.color="black",nodeNames = mynames, legend = TRUE)

**Step 3: Estimate the network structure using Bootnet**

mynetwork2<- bootnet_mgm(BPD_and_dissociation, type, level, tuning = 0.25, missing = c("listwise"), verbose = TRUE, criterion = c("EBIC"), nFolds = 10, order = 2, rule = c("AND"), unlock = TRUE, transform = c("none"))

**Step 4: Obtaining the Centrality Table, Centrality Plot, Network Plot and the Edge Weighting Table**

1. CentralityTable<- centralityTable(mynetwork2)

write.csv(CentralityTable, "MyCentralityTable.csv")

1. pdf("MyCentrality.pdf", width=4)

c1<- centralityPlot(myplot)

dev.off()

1. pdf("MyExpectedInfluence.pdf", width=4)

c2<- centralityPlot(myplot, include = "ExpectedInfluence")

dev.off()

1. int24<- showInteraction(object=mynetwork, int=c(2,3))

int24$parameters

round(mynetwork$pairwise$wadj, 2)

showInteraction(object = mynetwork,

int = c(8,9))

**Step 5: Bootstrapping the network structure**

b1<- bootnet(mynetwork2, boots=1000,nCores=8, statistics = c("strength","expectedInfluence","edge"))

b2<- bootnet(mynetwork2,noots=1000,nCores=4,type="case", statistics = c("strength","expectedInfluence","edge"))

**Step 6: Obtaining the Correlation Stability Coefficient**

corStability(b2)

pdf("EdgeStability.pdf")

plot(b1,labels = FALSE, order="sample")

dev.off()

pdf("CentrStability.pdf")

plot(b2)

dev.off()

**Step 7: Obtaining the Edge Weight Stability Plot**

plot(b1, labels = FALSE, order = "sample")

**Step 8: Obtaining the nodewise accuracy and the intercept/marginal model accuracy coefficient**

pred_obj <- predict(object = mynetwork,

data = BPD_and_dissociation,

errorCon = 'R2')

pred_obj$error

res_obj<- resample(object = mynetwork, data=as.matrix(BPD_and_dissociation), nB=50)

plotRes(object = res_obj,

quantiles = c(0.05, .95))

**Step 9. Obtaining the non-parametric difference test**

differenceTest(b1, "x", "y", "strength", alpha=0.05, verbose = TRUE)

plot(b1, "edge", plot = "difference", onlyNonZero = TRUE, order = "sample")

plot(b1, "strength", plot = "difference", onlyNonZero = FALSE, order = "sample")
